# Changing composition of SARS-CoV-2 lineages and rise of Delta variant in England

**DOI:** 10.1016/j.eclinm.2021.101064

**Published:** 2021-07-31

**Authors:** Swapnil Mishra, Sören Mindermann, Mrinank Sharma, Charles Whittaker, Thomas A Mellan, Thomas Wilton, Dimitra Klapsa, Ryan Mate, Martin Fritzsche, Maria Zambon, Janvi Ahuja, Adam Howes, Xenia Miscouridou, Guy P Nason, Oliver Ratmann, Elizaveta Semenova, Gavin Leech, Julia Fabienne Sandkühler, Charlie Rogers-Smith, Michaela Vollmer, H Juliette T Unwin, Yarin Gal, Meera Chand, Axel Gandy, Javier Martin, Erik Volz, Neil M Ferguson, Samir Bhatt, Jan M Brauner, Seth Flaxman, The COVID-19 Genomics UK (COG-UK) Consortium

**Affiliations:** a Medical Research Council (MRC) Centre for Global Infectious Disease Analysis, Jameel Institute, School of Public Health, Imperial College London, UK; b Oxford Applied and Theoretical Machine Learning (OATML) Group, Department of Computer Science, University of Oxford, UK; c Department of Statistics, University of Oxford, UK; d Department of Engineering Science, University of Oxford, UK; e Future of Humanity Institute, University of Oxford, UK; f National Institute for Biological Standards and Control (NIBSC), UK; g Public Health England, London, UK; h Medical Sciences Division, University of Oxford, UK; i Department of Mathematics, Imperial College London, UK; j Department of Computer Science, University of Bristol, UK; k Department of Psychology, University of Bonn, Germany; l OATML Group (work done while at OATML as an external collaborator), Department of Computer Science, University of Oxford, UK; m Section of Epidemiology, Department of Public Health, University of Copenhagen, Denmark

**Keywords:** SARS-CoV-2, Variants of concern, Epidemiology, Waste water monitoring, Genomic surveillance, Public health

## Abstract

**Background:**

Since its emergence in Autumn 2020, the SARS-CoV-2 Variant of Concern (VOC) B.1.1.7 (WHO label Alpha) rapidly became the dominant lineage across much of Europe. Simultaneously, several other VOCs were identified globally. Unlike B.1.1.7, some of these VOCs possess mutations thought to confer partial immune escape. Understanding when and how these additional VOCs pose a threat in settings where B.1.1.7 is currently dominant is vital.

**Methods:**

We examine trends in the prevalence of non-B.1.1.7 lineages in London and other English regions using passive-case detection PCR data, cross-sectional community infection surveys, genomic surveillance, and wastewater monitoring. The study period spans from 31st January 2021 to 15th May 2021.

**Findings:**

Across data sources, the percentage of non-B.1.1.7 variants has been increasing since late March 2021. This increase was initially driven by a variety of lineages with immune escape. From mid-April, B.1.617.2 (WHO label Delta) spread rapidly, becoming the dominant variant in England by late May.

**Interpretation:**

The outcome of competition between variants depends on a wide range of factors such as intrinsic transmissibility, evasion of prior immunity, demographic specificities and interactions with non-pharmaceutical interventions. The presence and rise of non-B.1.1.7 variants in March likely was driven by importations and some community transmission. There was competition between non-B.1.17 variants which resulted in B.1.617.2 becoming dominant in April and May with considerable community transmission. Our results underscore that early detection of new variants requires a diverse array of data sources in community surveillance. Continued real-time information on the highly dynamic composition and trajectory of different SARS-CoV-2 lineages is essential to future control efforts

**Funding:**

National Institute for Health Research, Medicines and Healthcare products Regulatory Agency, DeepMind, EPSRC, EA Funds programme, Open Philanthropy, Academy of Medical Sciences Bill,Melinda Gates Foundation, Imperial College Healthcare NHS Trust, The Novo Nordisk Foundation, MRC Centre for Global Infectious Disease Analysis, Community Jameel, Cancer Research UK, Imperial College COVID-19 Research Fund, Medical Research Council, Wellcome Sanger Institute.

**Research in Context**

**Evidence before this study**

Evidence about novel SARS-CoV-2 variants is rapidly being disseminated through genome sequencing databases, governmental reports, preprints, scientific papers, and even social media. We consulted journal publications, preprint repositories (medrxiv and biorxiv), and technical briefings from public health agencies (primarily Public Health England). For England, the COVID-19 Genomics UK Consortium (co-authors on this paper) maintain the most comprehensive dataset on genomic sequences. Data on variants obtained from genome sequencing databases often comes from non-random samples, meaning that there is a risk of bias. We also rely on estimates from the sequencing of viral RNA from sewage water. This form of environmental surveillance can be used to track variants with potentially lower bias due to the random sampling design.

**Added value of this study**

By bringing together passive-case detection PCR data, cross-sectional community infection surveys, genomic sequencing surveillance, and wastewater monitoring we are able to examine very recent spatial and temporal trends in the circulation of novel variants of SARS-CoV-2 in the regions of England. We highlight the situation that is currently unfolding in London where the pattern is clearest, and note similar patterns in other regions.

**Implications of all the available evidence**

We are witnessing dynamic shifts in the composition of SARS-CoV-2 lineages driving transmission across England in March and April 2021, with an expansion of non-B.1.1.7 VOCs. This still ambiguous but potentially concerning early signal of community transmission of non-B.1.1.7 VOCs in England suggests a need for intensified monitoring. Such information is critical to the epidemic's immediate control and to future vaccine development and deployment - both in the UK and other countries where the potential emergence of other novel SARS-CoV-2 variants remains a serious public health threat.

Alt-text: Unlabelled box

## 1 Introduction

Since its emergence in Autumn 2020 in South East England, the SARS-CoV-2 variant of concern (VOC) B.1.1.7 has become the dominant lineage across much of Europe [1]. Characterised by several mutations in the spike protein receptor-binding domain (RBD), epidemiological studies suggest B.1.1.7 is 50–80% more transmissible [2,3] and causes more severe disease [4] than previously circulating lineages. B.1.1.7 rose rapidly, from near 0% to over 50% in under two months, and soon made up >98% of sequenced samples in England. Its rapid spread necessitated a third English national lockdown in January 2021. Subsequent spread in Europe [5] and North America [6] has similarly highlighted the threat this variant poses to continued control of community transmission.

The 69–70 deletion in B.1.1.7′s Spike gene causes PCR tests to return negative results for that gene target [3], allowing S-gene target failure (SGTF) to act as a proxy for genomic surveillance. The rapidity of PCR testing means that this proxy is available more quickly than genomic sequencing data. Both community-based testing of symptomatic individuals (“Pillar 2″ [7]) and a weekly survey of more than 100,000 randomly sampled UK residents conducted by the Office for National Statistics (ONS) [8] have shown trends in SGTF frequency which mirrored the pattern seen in sequenced samples. The frequency of SGTF increased from near 0% in October 2020 to 98.8% in March 2021.

After B.1.1.7′s emergence, several other VOCs have been identified globally, including B.1.351 (first identified in South Africa [9]), P.1 (first identified in Brazil [10]), and B.1.617.2 (first identified in India). These VOCs have been associated with extensive transmission following emergence, leading to substantial infection and mortality rates even in settings where seroprevalence was high (for example in Manaus, Brazil [11,12]). Epidemiological analysis suggests that B.1.351 and P.1 are more transmissible than ancestral SARS-CoV-2 lineages; [10,13] for B.1.617.2, emerging evidence suggests the same. Additionally, all three VOCs carry mutations thought to contribute to partial immune escape (E484K or T478K) [[14], [15], [16]]. The three VOCs do not have the 69–70 deletion and can thus be distinguished from B.1.1.7 in the Spike gene PCR.

The UK now has a high level of population immunity to SARS-CoV-2: at the beginning of April 2021, it was estimated that 55% (95% CI: 49%−60%) of the English population were seropositive, either due to prior infection or vaccination [17]. However, such high levels of immunity also represent an evolutionary selection pressure on the virus and may give VOCs with even a partial degree of immune escape (relative to B.1.1.7) a transmission fitness advantage –especially at a time where control measures are being progressively relaxed. Further, the UK's vaccination rollout has relied heavily on the AstraZeneca vaccine; a vaccine that has proven highly protective against B.1.1.7 and prior variants [18], but may possess reduced efficacy against other VOCs [15]. Understanding when, how and if these VOCs pose a threat in settings where B.1.1.7 is currently dominant is vital also for other countries.

Here, we use a combination of data from passive-case detection PCR data, cross-sectional community infection surveys, genomic sequencing surveillance, and wastewater monitoring to examine spatial and temporal trends in the prevalence of non-B.1.1.7 lineages in England between February and May 2021.

## 2 Methods

### 2.1 Pillar 2 symptomatic community testing

Public Health England's surveillance system assembles data from dozens of PCR testing laboratories, the largest of which are the three large “Lighthouse” laboratories developed specifically in response to the pandemic. Approximately 30% of the samples processed by the Lighthouse laboratories use the ThermoFisher TaqPath PCR assay, which includes Spike as a target. For tests that give a PCR cycle threshold (Ct) value for non-spike targets substantially below the positivity threshold of 40, SGTF is a highly accurate proxy for B.1.1.7. Thus we are able to categorise a substantial proportion of all lab-confirmed community SARS-CoV cases as B.1.1.7 or non-B.1.1.7 [2]. SGTF becomes less reliable when Ct values for all targets are high since the Spike target is more likely to test negative by chance when sample viral load is low. Hence we estimate the frequency of SGTF only from cases with Ct values in non-Spike targets of 30 or less. However, results and conclusions were unchanged when we included cases with Ct of 40 or less.

We consider the period from 31st January 2021 to 15th May 2021. We only consider test results in self-reported symptomatic cases and exclude tests conducted following a lateral flow test (used, for instance, for asymptomatic screening for infection in schools and workplaces). Unlike the COG-UK data detailed below, we do not have metadata to exclude individuals with recent travel history. Over that period and with these exclusions applied, there was a total of 72,881 S-gene positive (S+), and 586,854 S-gene negative (S-) cases in England processed by the Lighthouse laboratories and 4246 S+ and 79,207 S- cases in London. Given that SGTF results are only available for a subset of samples, we estimate total Spike-positive (S+) case incidence by multiplying the frequency of S+ among all cases with SGTF results by the total Pillar 2 case incidence. Uncertainty estimates are detailed in Supplementary Text.

### 2.2 ONS infection survey

**Fig. 1:**
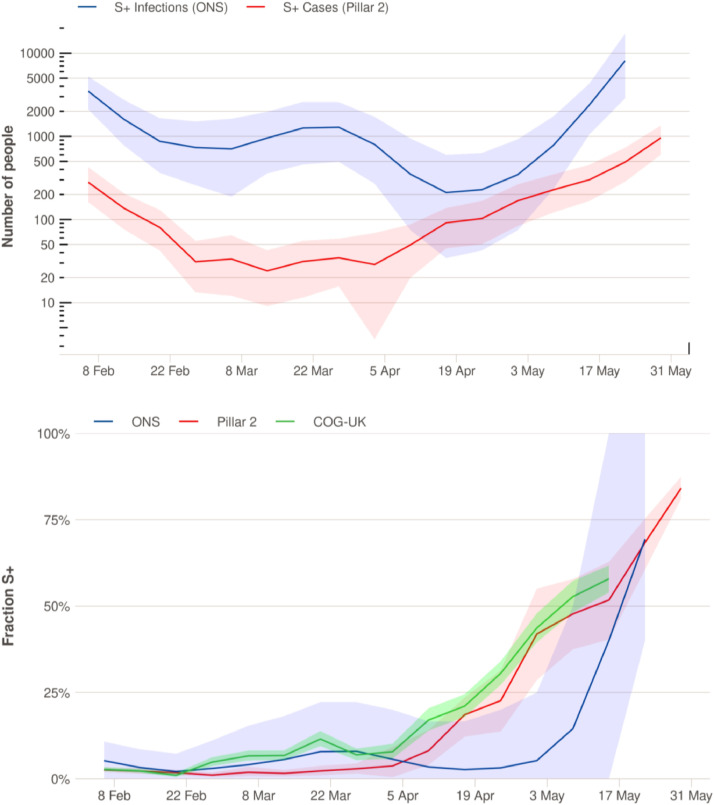
Trends in S+ infections in London, February-May 2021. (**A)** Estimated aggregated weekly incidence (log scale) of symptomatic S+ cases diagnosed via community testing (Pillar 2) calculated by multiplying the fraction of S+ cases by the total number of positives and S+ infections estimated from the ONS infection survey [35]. **B)** Temporal trends in the proportion of cases and infections that are S+, estimated from symptomatic community testing (Pillar 2), the ONS infection survey, and from SARS-CoV-2 sequence data (COG-UK public data, which may include travelers and surge testing; non-B.1.17 fraction is shown). Shaded ribbons represent 95% uncertainty intervals for the mean. Details on uncertainty intervals can be found in Supplementary Text. Results for other regions of England can be found in Supplementary Figures 1 and 2.

ONS conducts a fortnightly survey of randomly selected private households in the UK. In the two weeks prior to 16th April 2021, 139,948 participants from 73,328 households were tested using nose and throat self-swabs, analyzed with a PCR test. A Bayesian model was used to estimate the positivity rate for SARS-CoV-2 in the community, stratified by regions of England [19]. We use the ONS estimates of the percentage of PCR-positive samples that are “not compatible with UK variant” (gene pattern S + ORF1ab + N; indicated as S+ in Fig. 1) and the estimates of samples that are “UK variant compatible” (gene pattern ORF1ab + N indicating likely infection with B.1.1.7). Uncertainty estimates are detailed in Supplementary Text.

Each ONS release provides estimates for a 6 week period. We combine all the ONS releases from 26th February 2021 to 14th May 2021. For duplicated dates, we take the most recent estimate available in the combined data. To estimate total infection prevalence for each region (Fig. 1A and Supp Figure A), we multiply the estimated S+ infection prevalence for that region by its population size as reported by ONS [20].

### 2.3 Sewage water monitoring

Sequencing of viral RNA from sewage water has been a valuable tool for tracking the distribution of SARS-CoV-2 variants in the UK, both during the first wave [21] and the rise of B.1.1.7 [22]. In particular, a key advantage of this method is low sampling bias as it captures all people in the catchment area and not only those that receive COVID-19 tests. Here, we analysed fortnightly samples from the Beckton Sewage Treatment Works plant, which has a catchment area containing approximately 4 million people in North London. The catchment area does not include Heathrow Airport and adjacent quarantine hotels, which drain into the Mogden Sewage Treatment Works plant (as confirmed by Thames Water). Sample collection, processing, and analysis are described in detail in previous work; [21,22] a short summary is given in Supplementary Text.

### 2.4 COG-UK genomic sequencing

We studied 10,3247 sequences collected from Pillar 2 testing in the greater London area after March 1, 2021 and provided by the COG-UK consortium [23]. Sequence quality control, alignment, and lineage classification was carried out as described in previous work [24] and computed with the MRC—CLIMB computational infrastructure [25]. Among the 10,324 sequences, 2957 were found to be from a lineage other than B.1.1.7 with 2560 sequences in the set of VOCs and 397 variants under investigation (VUIs) P.1 (*n* = 81), B.1.1.318 (*n* = 74), B.1.525 (*n* = 96), B.1.617.2 (*n* = 2225), B.1.617.1 (*n* = 131), B.1.351 (*n* = 254) and C.36.3 (*n* = 28).

We estimated the frequency over time for each lineage with more than 20 samples using a Gaussian process generalized additive model with a multinomial response for each lineage (details are in Supplementary Text). Only a minority of the non-B.1.1.7 sequences (*n* = 2957) were found to be collected from managed quarantine facilities and individuals with recent travel history. We repeated the analysis excluding this set.

### 2.5 Statistical analysis

All analysis was done using R version 4.0.5 unless stated otherwise. We used a bootstrapping approach to obtain confidence intervals for Pillar 2 and COG-UK data. For sewage data the sequences were processed and analysed using Geneious 10.2.3 software. Statistical analysis of COG-UK sequencing data was performed using the MGCV package in R. See Supplementary Text: Methods for detailed description of the statistical methodologies we used.

### 2.6 Ethical approval

The COVID-19 Genomics UK Consortium has been given approval by Public Health England's Research Ethics and Governance Group (PHE R&D Ref: NR0195). For sewage data not applicable as no human materials were used in the study, and hence, no individual patient consent is required.

### 2.7 Reporting

The reporting of this paper adheres to the Strengthening the Reporting of Observational Studies in Epidemiology (STROBE)-guidelines.

### 2.8 Role of the funding source

Beyond supporting our work over the long term, no funding agency had any role in the study or its analysis.

## 3 Results

Since the beginning of March 2021, S+ case incidence (Pillar 2) has been increasing against a backdrop of initially falling, and then stable, low overall case numbers. Fig. 1 displays the data for London, where this trend started earliest, but similar increases in S+ cases happened in every other region in England (Supplementary Figs. 1 and 2). However, Pillar 2 is based on non-random testing. S+ infection prevalence (ONS) showed an early slight increase in March but then decreased again and increased strongly only in early May. However, the ONS survey suffers from sampling variability due to the low overall incidence in London. Similar patterns, with increases in the ONS survey lagging behind Pillar 2 data, are seen in several other regions of England (Supplementary Figs. 1 and 2).

**Fig. 2:**
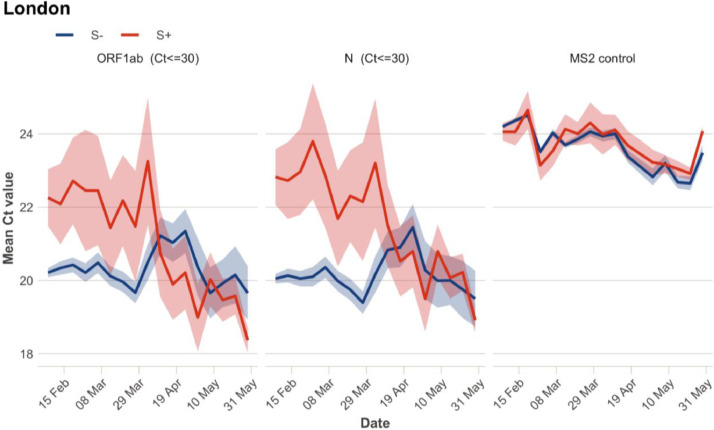
Mean Cycle threshold (Ct) values by week for Pillar 2 symptomatic community testing in London. Shaded ribbons show 95% confidence intervals around the mean calculated as 1.96 * standard error (assuming asymptotic normality). Ct values for ORF1ab gene and N gene are shown, with S+ in blue and S- in red. MS2 control indicates the mean Ct value of Bacteriophage MS2, which is added to samples for calibration purposes. In each plot, samples with Ct values above 30 for the specific gene shown are excluded. Results for other regions of England can be found in Supplementary Fig. 3.

Examination of the Pillar 2 Ct values supports a quantitative and qualitative change in S+ transmission patterns. Ct values in community testing are inversely related to viral load. Recent work has shown that population-level average Ct values can therefore provide an indication about the epidemic's dynamics, with average Ct values declining when epidemics are growing and increasing when epidemics are declining [26]. Fig. 2 shows that until March 2021, S- samples (primarily B.1.1.7) had considerably lower Ct values than S+ samples, especially for the N gene. This is as expected; reports suggest B.1.1.7 has higher viral loads, and thus lower Ct values, than prior lineages [27]. Since the end of March 2021, however, mean Ct values for S+ samples have considerably decreased and are now comparable to values for S- samples. This suggests an increase in transmission of S+ lineages; for imported cases, it may suggest an increasing epidemic in the country of origin. Additionally, a change in the genetic composition of S+ cases, towards variants causing higher viral loads, could also have contributed to the drop in Ct values.

**Fig. 3:**
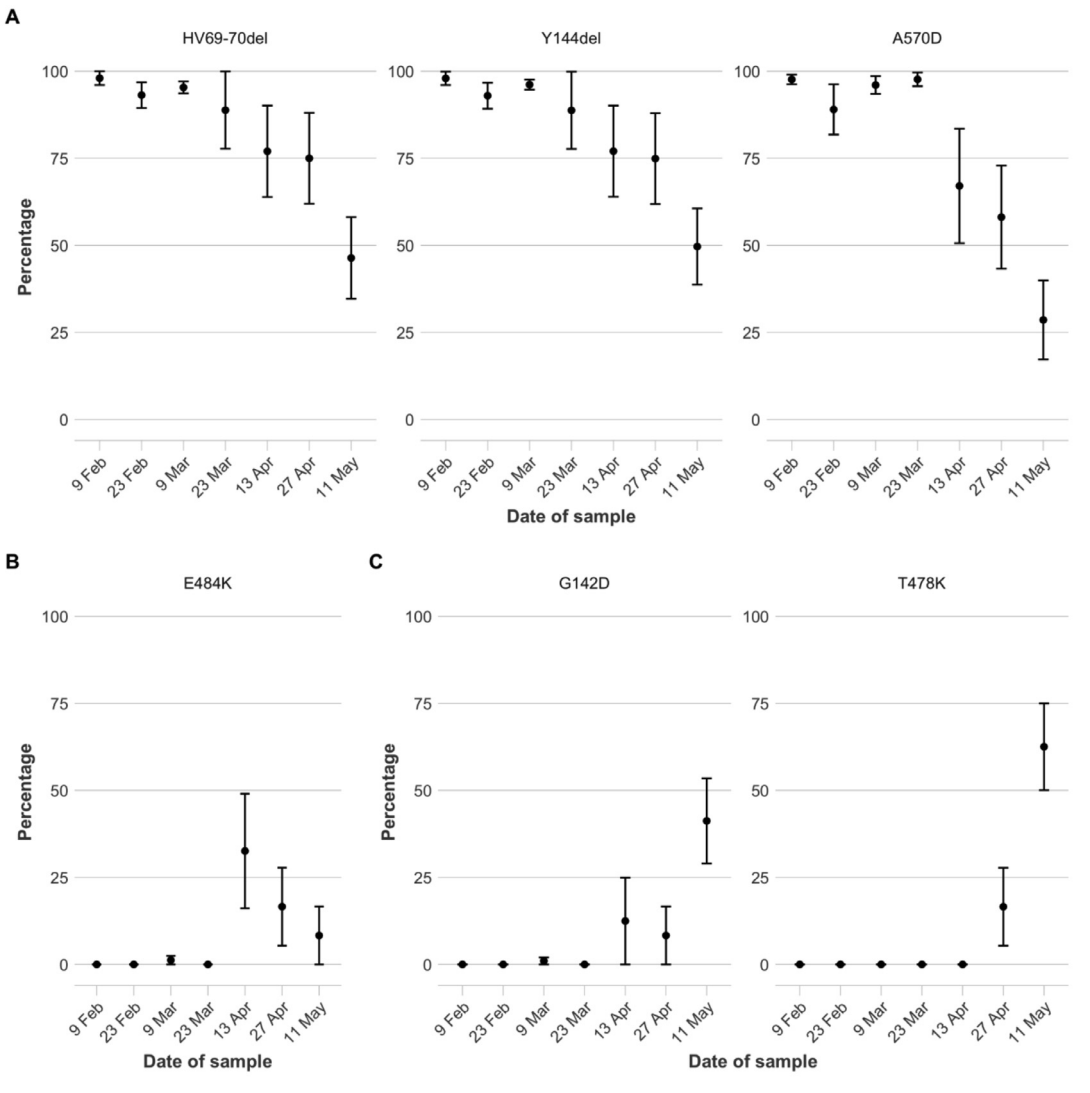
Fraction of viral RNA showing mutations at key spike protein amino acid positions, identified in sewage samples from North London. Mean values from replicate sequences (*n* = 8–12) for each sampling date are shown. Error bars indicate standard error of the mean. **A)** HV69–70del, Y144del, and A570D are relatively uniquely found in B.1.1.7 (Supplementary Table 1). **B)** E484K is absent in B.1.1.7. but present in several other variants of interest/concern; and linked to evasion of previous immunity. **C)** G142D and T478K are associated with B.1.617.2 (G142D is also found in B.1.617.1, Supplementary Table 1).

Fig. 3 shows the frequency of mutations in SARS-CoV-2 viral RNA found in sewage water [21,22] from North London. This data source includes all people living in the sewage plant's catchment area, not just those that are tested. Fig. 3 confirms that the increase in the proportion of S+ observed in other data sources is due to a decrease in the proportion of B.1.1.7, with mutations HV69–70del, Y144del, and A570D (all largely unique to B.1.1.7^22^; Supplementary Table 1) all showing considerable declines. All three mutations were detected at a stable frequency >95% from early January [22] to mid-March 2021 and then decreased to mean frequencies of 67% - 75% by April 13th (Fig. 3A). The frequency of the E484K mutation—absent in B.1.1.7 but present in many variants of concern that evade immunity—had increased to over 30% by April 13th, though it declined in the following weeks (Fig. 3B). The non-B.1.1.7 population on April 13th included variants B.1.351 and B.1.525 but not P.1 or B.1.617.2, as revealed by analysing additional mutations (Supplementary Text). After April 13th, B.1.1.7-associated mutations further decreased in frequency, to 28% - 49% by May 11th (Fig. 3A). In turn, B.1.617.2-associated mutations increased to 41% - 62% (Fig. 3C). In summary, sewage water samples suggest that various immunity-evading variants started to replace B.1.1.7 in the North London viral population by early April 2021. By mid-May a single variant of concern, B.1.617.2, dominated, constituting around half of the virus found in sewage water.

**Fig. 4:**
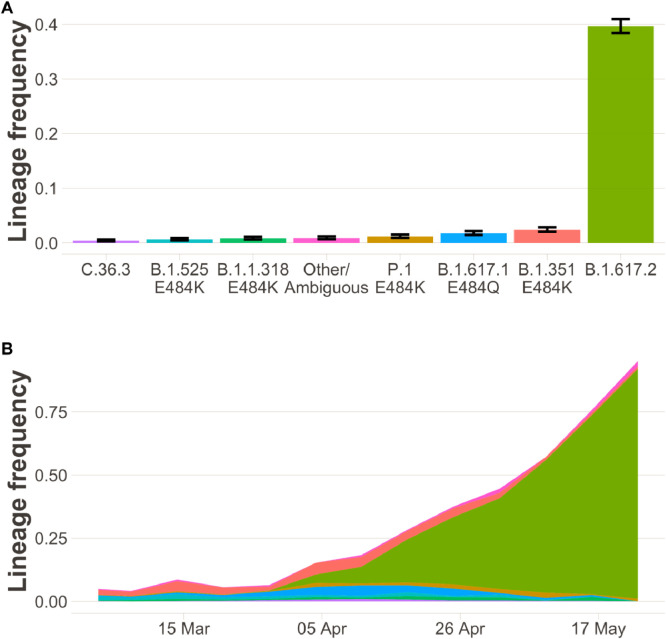
The sample frequency of non-B.1.1.7 lineages in Greater London in community testing (*n* = 2957 sequenced samples). (**A)** Bar charts show the sample proportion of lineages with at least 20 samples after 31 March 2021. Error bars show 95% confidence intervals based on binomial sampling. (**B)** Stacked area charts show estimates over time of the frequency of lineages in the period 1 March to 29 May. Colour-code is identical to panel A). While a variety of non-B.1.1.7 variants (all S+) are in circulation in March and the beginning of April, by May B.1.617.2 predominates. A of this figure, displaying data that was available until mid-April, can be found in the Supplement (Supplementary Fig. 6).

Fig. 4 shows results from COG-UK sequencing of SARS-CoV-2 samples from London, mirroring the sewage water results. Throughout March, the sequenced non-B.1.1.7 samples included chiefly B.1.351 and B.1.525 but also several other variants (see also Supplementary Figure 6). Over the course of April, the frequency of B.1.617.2 in sequenced samples increased rapidly, ultimately making up more than 75% of all sequences by late May. A similar overall pattern is seen when excluding cases which are linked to travel or surge-testing (Supplementary Figure 5), suggesting community transmission of B.1.617.2.

## 4 Discussion

Experiences across the globe to date have highlighted the significant public health threat that new SARS-CoV-2 VOCs can pose, even in settings where transmission is currently under control or where population-level immunity should preclude resurgence. They have also highlighted the importance of early detection and identification of emerging viral threats, which provides the opportunity for prompt implementation of measures to control spread. Here, using four independent data sources, we present evidence supporting recent increases in the number and proportion of COVID-19 infections that are S+; a dynamically changing population that was driven first by a variety of lineages with immune evasion (see below), and then overwhelmingly by the newest VOC, B.1.617.2.

When detecting increases in the proportion of a new variant, a key question is whether this reflects local transmission, or imported infections detected on the background of low overall incidence. Variants under investigation such as B.1.525 and A.23.1 have undergone periods of rapid expansion in January-March 2021 associated with travel-related importation and limited local spread, only to subside later. At the time of writing, however, B.1.617.2 has become the dominant variant in England (Supplementary Figures 1 and 2) and makes up more than 75% of sequenced samples in London even from cases which are not linked to travel (Supplementary Figure 5); sustained community transmission has taken place.

A considerable increase in the fraction of non-B.1.1.7 variants was apparent in multiple data sources already in early/mid-April 2021. This finding, and its consistency across independent data sources, gave an early warning about the potential for highly transmissible or immunity-evading variants to spread in England. However, data on the degree to which community transmission was driving this increase was ambiguous. While >20% of sequenced cases were from non-B.1.1.7 lineages as of mid-April, the fraction was only around 10% in cases not known to be associated with travel or surge testing (Supplementary Figure 6). There were clusters detected in London and elsewhere [9,28,29], but it was not known to what extent this transmission was self-sustaining or associated with short chains of transmission initiated by individual importation events. In addition, VOCs are subject to enhanced public health interventions, and thus the patterns seen in sequenced samples may deviate substantially from the overall population. Analysis of Ct values and mutations found in sewage water gave further evidence for community-transmission, but by no means conclusive. Sewage water sequencing is not subject to the same surveillance biases as symptomatic case testing, but the increase in non-B.1.1.7 variants in North London in April (Fig. 3) could still have been caused by an increase in imported infections, especially given that London has several large airports. Finally, decreasing Ct values (Fig. 2) can indicate rising epidemics, but they could also be explained by importation of infections from countries with rising epidemics.

Throughout May, the independent data sources we considered painted a consistent picture pointing to the rapid emergence and spread of B.1.617.2. S+ surveillance from Pillar 2–the most timely signal available due to the rapid turnaround of PCR testing–now serves as a useful proxy for B.1.617.2 vs B.1.1.7, due to the fact that B.1.617.2 now predominates among S+ variants, as confirmed by genomic surveillance of positive cases. While the ONS Infection Survey did not show signs of an increase in S+ in April (probably because the overall number of positive cases was very low), this population survey shows a marked increase in S+ in May, matching the other data streams. Finally, wastewater surveillance for North London is consistent with the rapid emergence of B.1.617.2 in April/May.

The outcome of competition between two variants depends on their relative transmission fitness, which is determined by the intrinsic transmissibility of each strain, the extent to which each can evade prior immunity, and any targeted non-pharmaceutical interventions in place. Several studies suggest that VOCs B.1.1.7 [2,3], P.1 [10], B.1.351^13^, and B.1.617.2^30^ are more transmissible than previously circulating lineages, but precise estimates of their relative transmissibility are not yet available. However, even if B.1.351, B.1.617.2 and P.1 are less intrinsically transmissible than B.1.1.7, any substantive ability to evade prior immunity may give these VOCs an overall transmission advantage over B.1.1.7 in the context of a highly immunised population such as the UK's. Mounting evidence from *in vitro* [*14*,*30*], epidemiological [10,13], and vaccine studies [15,16,31,32] suggests that variants with E484K, T478K, or E484Q mutations may partially evade prior immunity. Indeed, rapid resurgences followed variant emergence, for example in Manaus, Brazil (P.1) and Delhi, India (B.1.617.2), despite evidence of high levels of prior immunity in the population [11,33,34].

Events following the emergence of novel SARS-CoV-2 variants have emphasised the value of identifying and responding to changes in lineage frequency early. Our results underscore the value of utilising a diverse array of data sources in community surveillance. They also underscore the value of timely genomic surveillance to provide real-time information on the highly dynamic composition and trajectory of different SARS-CoV-2 lineages in a country. Such information is critical to the epidemic's immediate control and to future vaccine development and deployment - both in the UK and other countries where the potential emergence of other novel SARS-CoV-2 variants remains a serious public health threat.

## 5 Data sharing statement

Data underlying the figures, source code, and links to publicly available data sources can be found at https://github.com/ImperialCollegeLondon/SARS_CoV_2_variants_uk. Swapnil Mishra, Seth Flaxman, and Javier Martin are responsible for the veracity of and accessing the datasets used in this study.

## Funding

National Institute for Health Research, Medicines and Healthcare products Regulatory Agency, DeepMind, EPSRC, EA Funds programme, Open Philanthropy, Academy of Medical Sciences Bill,Melinda Gates Foundation, Imperial College Healthcare NHS Trust, The Novo Nordisk Foundation, MRC Centre for Global Infectious Disease Analysis, Community Jameel, Cancer Research UK, Imperial College COVID-19 Research Fund, Medical Research Council, Wellcome Sanger Institute.

## Contributors

Authors contributing to the formal analysis and design were S. Mishra, S. Flaxman, S. Bhatt, S. Mindermann, M. Sharma, J.M. Brauner, C. Whittaker, T.A. Mellan, E. Volz, J. Martin, N.M. Ferguson.

S. Mishra, S. Flaxman, S. Bhatt, S. Mindermann, M. Sharma, J.M. Brauner, C. Whittaker, N.M. Ferguson led the investigation and conceptualisation of the idea.

T. Wilton, D. Klapsa, R. Mate, M. Fritzsche, M. Zambon and J. Martin ran experiments and analysis for the sewage samples.

E. Volz and S. Flaxman analysed sequence data from COG-UK.

Swapnil Mishra, Seth Flaxman, and Javier Martin are responsible for the veracity of and accessing the datasets used in this study.

All authors contributed in writing and revising the manuscript.

## Declaration of Competing Interest

Dr. Semenova reports other from AstraZeneca, outside the submitted work;

M. Sharma reports grants from EPSRC Centre for Doctoral Training in Autonomous Intelligent Machines and Systems (EP/S024050/1) and a grant from the EA Funds programme, during the conduct of the study;

Dr. Martin reports grants from National Institute for Health Research (NIHR), during the conduct of the study; .

Dr. Nason reports and I am a member of the Royal Statistical Society's COVID-19 Taskforce.

Dr. Wilton reports grants from National Institute for Health Research (NIHR), during the conduct of the study; .

Dr. Mate reports grants from National Institute for Health Research (NIHR), during the conduct of the study; .

Dr. Klapsa reports grants from National Institute for Health Research (NIHR), during the conduct of the study; .

C. Rogers-Smith reports grants from Open Philanthropy, during the conduct of the study; .

Dr. Gal reports grants from research grant (studentship) from GlaxoSmithKline, outside the submitted work; .

Dr. Brauner reports grants from Cancer Research UK, during the conduct of the study;

Dr. Ferguson reports grants from UK Medical Research Council, grants from UK National Institute of Health Research, grants from Community Jameel, during the conduct of the study; grants from NIH NIGMS, grants from Janssen Pharmaceuticals, grants from Bill and Melinda Gates Foundation, grants from Gavi, the Vaccine Alliance, outside the submitted work;

All other authors declare no conflicts of interests or competing interests.

## Appendix Supplementary materials

Image, application 1
