# Supplementary material for: Changing composition of SARS-CoV-2 lineages and rise of Delta variant in England
Source: eClinicalMedicine. 2021 Jul 31;39:101064. doi: 10.1016/j.eclinm.2021.101064 (PMC8349999; doi:10.1016/j.eclinm.2021.101064)
Supplement: Supplementary file 1 [file mmc1.docx]

| Cherian Koshy^1^ |
| --- |
| Amy Ash^1^ |
| Emma Wise^2^ |
| Nathan Moore^2^ |
| Matilde Mori^2^ |
| Nick Cortes^2^ |
| Jessica Lynch^2^ |
| Stephen Kidd^2^ |
| Derek J Fairley^3^ |
| Tanya Curran^3^ |
| James P McKenna^3^ |
| Helen Adams^4^ |
| Christophe Fraser^5^ |
| Tanya Golubchik^5^ |
| David Bonsall^5^ |
| Mohammed O Hassan-Ibrahim^6^ |
| Cassandra S Malone^6^ |
| Benjamin J Cogger^6^ |
| Michelle Wantoch^7^ |
| Nicola Reynolds^7^ |
| Ben Warne^8^ |
| Joshua Maksimovic^9^ |
| Karla Spellman^9^ |
| Kathryn McCluggage^9^ |
| Michaela John^9^ |
| Robert Beer^9^ |
| Safiah Afifi^9^ |
| Sian Morgan^9^ |
| Angela Marchbank^10^ |
| Anna Price^10^ |
| Christine Kitchen^10^ |
| Huw Gulliver^10^ |
| Ian Merrick^10^ |
| Joel Southgate^10^ |
| Martyn Guest^10^ |
| Robert Munn^10^ |
| Trudy Workman^10^ |
| Thomas R Connor^10^ |
| William Fuller^10^ |
| Catherine Bresner^10^ |
| Luke B Snell^11^ |
| Amita Patel^11^ |
| Themoula Charalampous^12^ |
| Gaia Nebbia^12^ |
| Rahul Batra^12^ |
| Jonathan Edgeworth^12^ |
| Samuel C Robson^13^ |
| Angela H Beckett^13^ |
| David M Aanensen^14^ |
| Anthony P Underwood^14^ |
| Corin A Yeats^14^ |
| Khalil Abudahab^14^ |
| Ben EW Taylor^14^ |
| Mirko Menegazzo^14^ |
| Gemma Clark^15^ |
| Wendy Smith^15^ |
| Manjinder Khakh^15^ |
| Vicki M Fleming^15^ |
| Michelle M Lister^15^ |
| Hannah C Howson-Wells^15^ |
| Louise Berry^15^ |
| Tim Boswell^15^ |
| Amelia Joseph^15^ |
| Iona Willingham^15^ |
| Carl Jones^15^ |
| Christopher Holmes^16^ |
| Paul Bird^16^ |
| Thomas Helmer^16^ |
| Karlie Fallon^16^ |
| Julian Tang^16^ |
| Veena Raviprakash^17^ |
| Sharon Campbell^17^ |
| Nicola Sheriff^17^ |
| Victoria Blakey^17^ |
| Lesley-Anne Williams^17^ |
| Matthew W Loose^18^ |
| Nadine Holmes^18^ |
| Christopher Moore^18^ |
| Matthew Carlile^18^ |
| Victoria Wright^18^ |
| Fei Sang^18^ |
| Johnny Debebe^18^ |
| Francesc Coll^19^ |
| Adrian W Signell^20^ |
| Gilberto Betancor^20^ |
| Harry D Wilson^20^ |
| Sahar Eldirdiri^21^ |
| Anita Kenyon^21^ |
| Thomas Davis^21^ |
| Oliver G Pybus^22^ |
| Louis du Plessis^22^ |
| Alex E Zarebski^22^ |
| Jayna Raghwani^22^ |
| Moritz UG Kraemer^22^ |
| Sarah Francois^22^ |
| Stephen W Attwood^22^ |
| Tetyana I Vasylyeva^22^ |
| Marina Escalera Zamudio^22^ |
| Bernardo Gutierrez^22^ |
| M. Estee Torok^23^ |
| William L Hamilton^23^ |
| Ian G Goodfellow^24^ |
| Grant Hall^24^ |
| Aminu S Jahun^24^ |
| Yasmin Chaudhry^24^ |
| Myra Hosmillo^24^ |
| Malte L Pinckert^24^ |
| Iliana Georgana^24^ |
| Samuel Moses^25^ |
| Hannah Lowe^25^ |
| Luke Bedford^26^ |
| Jonathan Moore^27^ |
| Susanne Stonehouse^27^ |
| Chloe L Fisher^28^ |
| Ali R Awan^28^ |
| John BoYes^29^ |
| Judith Breuer^30^ |
| Kathryn Ann Harris^30^ |
| Julianne Rose Brown^30^ |
| Divya Shah^30^ |
| Laura Atkinson^30^ |
| Jack CD Lee^30^ |
| Nathaniel Storey^30^ |
| Flavia Flaviani^31^ |
| Adela Alcolea-Medina^32^ |
| Rebecca Williams^33^ |
| Gabrielle Vernet^33^ |
| Michael R Chapman^34^ |
| Lisa J Levett^35^ |
| Judith Heaney^35^ |
| Wendy Chatterton^35^ |
| Monika Pusok^35^ |
| Li Xu-McCrae^36^ |
| Darren L Smith^37^ |
| Matthew Bashton^37^ |
| Gregory R Young^37^ |
| Alison Holmes^38^ |
| Paul Anthony Randell^38^ |
| Alison Cox^38^ |
| Pinglawathee Madona^38^ |
| Frances Bolt^38^ |
| James Price^38^ |
| Siddharth Mookerjee^38^ |
| Manon Ragonnet-Cronin^39^ |
| Fabricia F. Nascimento^39^ |
| David Jorgensen^39^ |
| Igor Siveroni^39^ |
| Rob Johnson^39^ |
| Olivia Boyd^39^ |
| Lily Geidelberg^39^ |
| Erik M Volz^39^ |
| Aileen Rowan^39^ |
| Graham P Taylor^39^ |
| Katherine L Smollett^40^ |
| Nicholas J Loman^41^ |
| Joshua Quick^41^ |
| Claire McMurray^41^ |
| Joanne Stockton^41^ |
| Sam Nicholls^41^ |
| Will Rowe^41^ |
| Radoslaw Poplawski^41^ |
| Alan McNally^41^ |
| Rocio T Martinez Nunez^42^ |
| Jenifer Mason^43^ |
| Trevor I Robinson^43^ |
| Elaine O'Toole^43^ |
| Joanne Watts^43^ |
| Cassie Breen^43^ |
| Angela Cowell^43^ |
| Graciela Sluga^44^ |
| Nicholas W Machin^45^ |
| Shazaad S Y Ahmad^45^ |
| Ryan P George^45^ |
| Fenella Halstead^46^ |
| Venkat Sivaprakasam^46^ |
| Wendy Hogsden^46^ |
| Chris J Illingworth^47^ |
| Chris Jackson^47^ |
| Emma C Thomson^48^ |
| James G Shepherd^48^ |
| Patawee Asamaphan^48^ |
| Marc O Niebel^48^ |
| Kathy K Li^48^ |
| Rajiv N Shah^48^ |
| Natasha G Jesudason^48^ |
| Lily Tong^48^ |
| Alice Broos^48^ |
| Daniel Mair^48^ |
| Jenna Nichols^48^ |
| Stephen N Carmichael^48^ |
| Kyriaki Nomikou^48^ |
| Elihu Aranday-Cortes^48^ |
| Natasha Johnson^48^ |
| Igor Starinskij^48^ |
| Ana da Silva Filipe^48^ |
| David L Robertson^48^ |
| Richard J Orton^48^ |
| Joseph Hughes^48^ |
| Sreenu Vattipally^48^ |
| Joshua B Singer^48^ |
| Seema Nickbakhsh^48^ |
| Antony D Hale^49^ |
| Louissa R Macfarlane-Smith^49^ |
| Katherine L Harper^49^ |
| Holli Carden^49^ |
| Yusri Taha^50^ |
| Brendan AI Payne^50^ |
| Shirelle Burton-Fanning^50^ |
| Sheila Waugh^50^ |
| Jennifer Collins^50^ |
| Gary Eltringham^50^ |
| Steven Rushton^51^ |
| Sarah O'Brien^51^ |
| Amanda Bradley^52^ |
| Alasdair Maclean^52^ |
| Guy Mollett^52^ |
| Rachel Blacow^52^ |
| Kate E Templeton^53^ |
| Martin P McHugh^53^ |
| Rebecca Dewar^53^ |
| Elizabeth Wastenge^53^ |
| Samir Dervisevic^54^ |
| Rachael Stanley^54^ |
| Emma J Meader^54^ |
| Lindsay Coupland^54^ |
| Louise Smith^55^ |
| Clive Graham^56^ |
| Edward Barton^56^ |
| Debra Padgett^56^ |
| Garren Scott^56^ |
| Emma Swindells^57^ |
| Jane Greenaway^57^ |
| Andrew Nelson^58^ |
| Clare M McCann^58^ |
| Wen C Yew^58^ |
| Monique Andersson^59^ |
| Timothy Peto^59^ |
| Anita Justice^59^ |
| David Eyre^59^ |
| Derrick Crook^59^ |
| Tim J Sloan^60^ |
| Nichola Duckworth^60^ |
| Sarah Walsh^60^ |
| Anoop J Chauhan^61^ |
| Sharon Glaysher^61^ |
| Kelly Bicknell^61^ |
| Sarah Wyllie^61^ |
| Scott Elliott^61^ |
| Allyson Lloyd^61^ |
| Robert Impey^61^ |
| Nick Levene^62^ |
| Lynn Monaghan^62^ |
| Declan T Bradley^63^ |
| Tim Wyatt^63^ |
| Elias Allara^64^ |
| Clare Pearson^64^ |
| Husam Osman^64^ |
| Andrew Bosworth^64^ |
| Esther Robinson^64^ |
| Peter Muir^64^ |
| Ian B Vipond^64^ |
| Richard Hopes^64^ |
| Hannah M Pymont^64^ |
| Stephanie Hutchings^64^ |
| Martin D Curran^65^ |
| Surendra Parmar^65^ |
| Angie Lackenby^66^ |
| Tamyo Mbisa^66^ |
| Steven Platt^66^ |
| Shahjahan Miah^66^ |
| David Bibby^66^ |
| Carmen Manso^66^ |
| Jonathan Hubb^66^ |
| Meera Chand^66^ |
| Gavin Dabrera^66^ |
| Mary Ramsay^66^ |
| Daniel Bradshaw^66^ |
| Alicia Thornton^66^ |
| Richard Myers^66^ |
| Ulf Schaefer^66^ |
| Natalie Groves^66^ |
| Eileen Gallagher^66^ |
| David Lee^66^ |
| David Williams^66^ |
| Nicholas Ellaby^66^ |
| Ian Harrison^66^ |
| Hassan Hartman^66^ |
| Nikos Manesis^66^ |
| Vineet Patel^66^ |
| Chloe Bishop^66^ |
| Vicki Chalker^66^ |
| Juan Ledesma^67^ |
| Katherine A Twohig^67^ |
| Matthew T.G. Holden^68^ |
| Sharif Shaaban^68^ |
| Alec Birchley^69^ |
| Alexander Adams^69^ |
| Alisha Davies^69^ |
| Amy Gaskin^69^ |
| Amy Plimmer^69^ |
| Bree Gatica-Wilcox^69^ |
| Caoimhe McKerr^69^ |
| Catherine Moore^69^ |
| Chris Williams^69^ |
| David Heyburn^69^ |
| Elen De Lacy^69^ |
| Ember Hilvers^69^ |
| Fatima Downing^69^ |
| Giri Shankar^69^ |
| Hannah Jones^69^ |
| Hibo Asad^69^ |
| Jason Coombes^69^ |
| Joanne Watkins^69^ |
| Johnathan M Evans^69^ |
| Laia Fina^69^ |
| Laura Gifford^69^ |
| Lauren Gilbert^69^ |
| Lee Graham^69^ |
| Malorie Perry^69^ |
| Mari Morgan^69^ |
| Matthew Bull^69^ |
| Michelle Cronin^69^ |
| Nicole Pacchiarini^69^ |
| Noel Craine^69^ |
| Rachel Jones^69^ |
| Robin Howe^69^ |
| Sally Corden^69^ |
| Sara Rey^69^ |
| Sara Kumziene-SummerhaYes^69^ |
| Sarah Taylor^69^ |
| Simon Cottrell^69^ |
| Sophie Jones^69^ |
| Sue Edwards^69^ |
| Justin O’Grady^70^ |
| Andrew J Page^70^ |
| Alison E Mather^70^ |
| David J Baker^70^ |
| Steven Rudder^70^ |
| Alp Aydin^70^ |
| Gemma L Kay^70^ |
| Alexander J Trotter^70^ |
| Nabil-Fareed Alikhan^70^ |
| Leonardo de Oliveira Martins^70^ |
| Thanh Le-Viet^70^ |
| Lizzie Meadows^70^ |
| Anna Casey^71^ |
| Liz Ratcliffe^71^ |
| David A Simpson^72^ |
| Zoltan Molnar^72^ |
| Thomas Thompson^72^ |
| Erwan Acheson^72^ |
| Jane AH Masoli^73^ |
| Bridget A Knight^73^ |
| Sian Ellard^73^ |
| Cressida Auckland^73^ |
| Christopher R Jones^73^ |
| Tabitha W Mahungu^74^ |
| Dianne Irish-Tavares^74^ |
| Tanzina Haque^74^ |
| Jennifer Hart^74^ |
| Eric Witele^74^ |
| Melisa Louise Fenton^75^ |
| Ashok Dadrah^75^ |
| Amanda Symmonds^75^ |
| Tranprit Saluja^75^ |
| Yann Bourgeois^76^ |
| Garry P Scarlett^76^ |
| Katie F Loveson^77^ |
| Salman Goudarzi^77^ |
| Christopher Fearn^77^ |
| Kate Cook^77^ |
| Hannah Dent^77^ |
| Hannah Paul^77^ |
| David G Partridge^78^ |
| Mohammad Raza^78^ |
| Cariad Evans^78^ |
| Kate Johnson^78^ |
| Steven Liggett^79^ |
| Paul Baker^79^ |
| Stephen Bonner^79^ |
| Sarah Essex^79^ |
| Ronan A Lyons^80^ |
| Kordo Saeed^81^ |
| Adhyana I.K Mahanama^81^ |
| Buddhini Samaraweera^81^ |
| Siona Silveira^81^ |
| Emanuela Pelosi NA^81^ |
| Eleri Wilson-Davies^81^ |
| Rachel J Williams^82^ |
| Mark Kristiansen^82^ |
| Sunando Roy^82^ |
| Charlotte A Williams^82^ |
| Marius Cotic^82^ |
| Nadua Bayzid^82^ |
| Adam P Westhorpe^82^ |
| John A Hartley^82^ |
| Riaz Jannoo^82^ |
| Helen L Lowe^82^ |
| Angeliki Karamani^82^ |
| Leah Ensell^82^ |
| Jacqui A Prieto^83^ |
| Sarah Jeremiah^83^ |
| Dimitris Grammatopoulos^84^ |
| Sarojini Pandey^84^ |
| Lisa Berry^84^ |
| Katie Jones^84^ |
| Alex Richter^85^ |
| Andrew Beggs^85^ |
| Angus Best^86^ |
| Benita Percival^86^ |
| Jeremy Mirza^86^ |
| Oliver Megram^86^ |
| Megan Mayhew^86^ |
| Liam Crawford^86^ |
| Fiona Ashcroft^86^ |
| Emma Moles-Garcia^86^ |
| Nicola Cumley^86^ |
| Colin P Smith^87^ |
| Giselda Bucca^87^ |
| Andrew R Hesketh^87^ |
| Beth Blane^88^ |
| Sophia T Girgis^88^ |
| Danielle Leek^88^ |
| Sushmita Sridhar^88^ |
| Sally Forrest^88^ |
| Claire Cormie^88^ |
| Harmeet K Gill^88^ |
| Joana Dias^88^ |
| Ellen E Higginson^88^ |
| Mailis Maes^88^ |
| Jamie Young^88^ |
| Leanne M Kermack^88^ |
| Ravi Kumar Gupta^88^ |
| Catherine Ludden^88^ |
| Sharon J Peacock^88^ |
| Sophie Palmer^88^ |
| Carol M Churcher^88^ |
| Nazreen F Hadjirin^88^ |
| Alessandro M Carabelli^88^ |
| Ellena Brooks^88^ |
| Kim S Smith^88^ |
| Katerina Galai^88^ |
| Georgina M McManus^88^ |
| Chris Ruis^88^ |
| Rose K Davidson^89^ |
| Andrew Rambaut^90^ |
| Thomas Williams^90^ |
| Carlos E Balcazar^90^ |
| Michael D Gallagher^90^ |
| Áine O'Toole^90^ |
| Stefan Rooke^90^ |
| Verity Hill^90^ |
| Kathleen A Williamson^90^ |
| Thomas D Stanton^90^ |
| Stephen L Michell^91^ |
| Claire M Bewshea^91^ |
| Ben Temperton^91^ |
| Michelle L Michelsen^91^ |
| Joanna Warwick-Dugdale^91^ |
| Robin Manley^91^ |
| Audrey Farbos^91^ |
| James W Harrison^91^ |
| Christine M Sambles^91^ |
| David J Studholme^91^ |
| Aaron R Jeffries^91^ |
| Leigh M Jackson^91^ |
| Alistair C Darby^92^ |
| Julian A Hiscox^92^ |
| Steve Paterson^92^ |
| Miren Iturriza-Gomara^92^ |
| Kathryn A Jackson^92^ |
| Anita O Lucaci^92^ |
| Edith E Vamos^92^ |
| Margaret Hughes^92^ |
| Lucille Rainbow^92^ |
| Richard Eccles^92^ |
| Charlotte Nelson^92^ |
| Mark Whitehead^92^ |
| Lance Turtle^92^ |
| Sam T Haldenby^92^ |
| Richard Gregory^92^ |
| Matthew Gemmell^92^ |
| Claudia Wierzbicki^92^ |
| Hermione J Webster^92^ |
| Thushan I de Silva^93^ |
| Nikki Smith^93^ |
| Adrienn Angyal^93^ |
| Benjamin B Lindsey^93^ |
| Danielle C Groves^93^ |
| Luke R Green^93^ |
| Dennis Wang^93^ |
| Timothy M Freeman^93^ |
| Matthew D Parker^93^ |
| Alexander J Keeley^93^ |
| Paul J Parsons^93^ |
| Rachel M Tucker^93^ |
| Rebecca Brown^93^ |
| Matthew Wyles^93^ |
| Max Whiteley^93^ |
| Peijun Zhang^93^ |
| Marta Gallis^93^ |
| Stavroula F Louka^93^ |
| Chrystala Constantinidou^94^ |
| Meera Unnikrishnan^94^ |
| Sascha Ott^94^ |
| Jeffrey K. J. Cheng^94^ |
| Hannah E. Bridgewater^94^ |
| Lucy R. Frost^94^ |
| Grace Taylor-Joyce^94^ |
| Richard Stark^94^ |
| Laura Baxter^94^ |
| Mohammad T. Alam^94^ |
| Paul E Brown^94^ |
| Dinesh Aggarwal^95^ |
| Alberto C Cerda^96^ |
| Tammy‎ V Merrill^96^ |
| Rebekah E Wilson^96^ |
| Patrick C McClure^97^ |
| Joseph G Chappell^97^ |
| Theocharis Tsoleridis^97^ |
| Jonathan Ball^97^ |
| David Buck^98^ |
| John A Todd^98^ |
| Angie Green^98^ |
| Amy Trebes^98^ |
| George MacIntyre-Cockett^98^ |
| Mariateresa de Cesare^98^ |
| Sanger Covid Team (www.sanger.ac.uk/covid-team) NA^99^ |
| Alex Alderton^99^ |
| Roberto Amato^99^ |
| Cristina V Ariani^99^ |
| Mathew A Beale^99^ |
| Charlotte Beaver^99^ |
| Katherine L Bellis^99^ |
| Emma Betteridge^99^ |
| James Bonfield^99^ |
| John Danesh^99^ |
| Matthew J Dorman^99^ |
| Eleanor Drury^99^ |
| Ben W Farr^99^ |
| Luke Foulser^99^ |
| Sonia Goncalves^99^ |
| Scott Goodwin^99^ |
| Marina Gourtovaia^99^ |
| Ewan M Harrison^99^ |
| David K Jackson^99^ |
| Dorota Jamrozy^99^ |
| Ian Johnston^99^ |
| Leanne Kane^99^ |
| Sally Kay^99^ |
| Jon-Paul Keatley^99^ |
| Dominic Kwiatkowski^99^ |
| Cordelia F Langford^99^ |
| Mara Lawniczak^99^ |
| Laura Letchford^99^ |
| Rich Livett^99^ |
| Stephanie Lo^99^ |
| Inigo Martincorena^99^ |
| Samantha McGuigan^99^ |
| Rachel Nelson^99^ |
| Steve Palmer^99^ |
| Naomi R Park^99^ |
| Minal Patel^99^ |
| Liam Prestwood^99^ |
| Christoph Puethe^99^ |
| Michael A Quail^99^ |
| Shavanthi Rajatileka^99^ |
| Carol Scott^99^ |
| Lesley Shirley^99^ |
| John Sillitoe^99^ |
| Michael H Spencer Chapman^99^ |
| Scott AJ Thurston^99^ |
| Gerry Tonkin-Hill^99^ |
| Danni Weldon^99^ |
| Diana Rajan^99^ |
| Iraad F Bronner^99^ |
| Louise Aigrain^99^ |
| Nicholas M Redshaw^99^ |
| Stefanie V Lensing^99^ |
| Robert Davies^99^ |
| Andrew Whitwham^99^ |
| Jennifier Liddle^99^ |
| Kevin Lewis^99^ |
| Jaime M Tovar-Corona^99^ |
| Steven Leonard^99^ |
| Jillian Durham^99^ |
| Andrew R Bassett^99^ |
| Shane McCarthy^99^ |
| Robin J Moll^99^ |
| Keith James^99^ |
| Karen Oliver^99^ |
| Alex Makunin^99^ |
| Jeff Barrett^99^ |
| Rory N Gunson^100^ |

| 1 Barking, Havering and Redbridge University Hospitals NHS Trust |
| --- |
| 2 Basingstoke Hospital |
| 3 Belfast Health & Social Care Trust |
| 4 Betsi Cadwaladr University Health Board |
| 5 Big Data Institute, Nuffield Department of Medicine, University of Oxford |
| 6 Brighton and Sussex University Hospitals NHS Trust |
| 7 Cambridge Stem Cell Institute, University of Cambridge |
| 8 Cambridge University Hospitals NHS Foundation Trust |
| 9 Cardiff and Vale University Health Board |
| 10 Cardiff University |
| 11 Centre for Clinical Infection & Diagnostics Research, St. Thomas' Hospital and Kings College London |
| 12 Centre for Clinical Infection and Diagnostics Research, Department of Infectious Diseases, Guy's and St Thomas' NHS Foundation Trust |
| 13 Centre for Enzyme Innovation, University of Portsmouth (PORT) |
| 14 Centre for Genomic Pathogen Surveillance, University of Oxford |
| 15 Clinical Microbiology Department, Queens Medical Centre |
| 16 Clinical Microbiology, University Hospitals of Leicester NHS Trust |
| 17 County Durham and Darlington NHS Foundation Trust |
| 18 Deep Seq, School of Life Sciences, Queens Medical Centre, University of Nottingham |
| 19 Department of Infection Biology, Faculty of Infectious & Tropical Diseases, London School of Hygiene & Tropical Medicine |
| 20 Department of Infectious Diseases, King's College London |
| 21 Department of Microbiology, Kettering General Hospital |
| 22 Department of Zoology, University of Oxford |
| 23 Departments of Infectious Diseases and Microbiology, Cambridge University Hospitals NHS Foundation Trust; Cambridge, UK |
| 24 Division of Virology, Department of Pathology, University of Cambridge |
| 25 East Kent Hospitals University NHS Foundation Trust |
| 26 East Suffolk and North Essex NHS Foundation Trust |
| 27 Gateshead Health NHS Foundation Trust |
| 28 Genomics Innovation Unit, Guy's and St. Thomas' NHS Foundation Trust |
| 29 Gloucestershire Hospitals NHS Foundation Trust |
| 30 Great Ormond Street Hospital for Children NHS Foundation Trust |
| 31 Guy's and St. Thomas’ BRC |
| 32 Guy's and St. Thomas’ Hospitals |
| 33 Hampshire Hospitals NHS Foundation Trust |
| 34 Health Data Research UK Cambridge |
| 35 Health Services Laboratories |
| 36 Heartlands Hospital, Birmingham |
| 37 Hub for Biotechnology in the Built Environment, Northumbria University |
| 38 Imperial College Hospitals NHS Trust |
| 39 Imperial College London |
| 40 Institute of Biodiversity, Animal Health & Comparative Medicine |
| 41 Institute of Microbiology and Infection, University of Birmingham |
| 42 King's College London |
| 43 Liverpool Clinical Laboratories |
| 44 Maidstone and Tunbridge Wells NHS Trust |
| 45 Manchester University NHS Fountation Trust |
| 46 Microbiology Department, Wye Valley NHS Trust, Hereford |
| 47 MRC Biostatistics Unit, University of Cambridge |
| 48 MRC-University of Glasgow Centre for Virus Research |
| 49 National Infection Service, PHE and Leeds Teaching Hospitals Trust |
| 50 Newcastle Hospitals NHS Foundation Trust |
| 51 Newcastle University |
| 52 NHS Greater Glasgow and Clyde |
| 53 NHS Lothian |
| 54 Norfolk and Norwich University Hospital |
| 55 Norfolk County Council |
| 56 North Cumbria Integrated Care NHS Foundation Trust |
| 57 North Tees and Hartlepool NHS Foundation Trust |
| 58 Northumbria University |
| 59 Oxford University Hospitals NHS Foundation Trust |
| 60 PathLinks, Northern Lincolnshire & Goole NHS Foundation Trust |
| 61 Portsmouth Hospitals University NHS Trust |
| 62 Princess Alexandra Hospital Microbiology Dept. |
| 63 Public Health Agency |
| 64 Public Health England |
| 65 Public Health England, Clinical Microbiology and Public Health Laboratory, Cambridge, UK |
| 66 Public Health England, Colindale |
| 67 Public Health England, Colindale |
| 68 Public Health Scotland |
| 69 Public Health Wales NHS Trust |
| 70 Quadram Institute Bioscience |
| 71 Queen Elizabeth Hospital |
| 72 Queen's University Belfast |
| 73 Royal Devon and Exeter NHS Foundation Trust |
| 74 Royal Free NHS Trust |
| 75 Sandwell and West Birmingham NHS Trust |
| 76 School of Biological Sciences, University of Portsmouth (PORT) |
| 77 School of Pharmacy and Biomedical Sciences, University of Portsmouth (PORT) |
| 78 Sheffield Teaching Hospitals |
| 79 South Tees Hospitals NHS Foundation Trust |
| 80 Swansea University |
| 81 Univeristy Hospitals Southampton NHS Foundation Trus |
| 82 University College London |
| 83 University Hospital Southampton NHS Foundation Trust |
| 84 University Hospitals Coventry and Warwickshire |
| 85 University of Birmingham |
| 86 University of Birmingham Turnkey Laboratory |
| 87 University of Brighton |
| 88 University of Cambridge |
| 89 University of East Anglia |
| 90 University of Edinburgh |
| 91 University of Exeter |
| 92 University of Liverpool |
| 93 University of Sheffield |
| 94 University of Warwick |
| 95 Univesity of Cambridge |
| 96 Viapath, Guy's and St Thomas' NHS Foundation Trust, and King's College Hospital NHS Foundation Trust |
| 97 Virology, School of Life Sciences, Queens Medical Centre, University of Nottingham |
| 98 Wellcome Centre for Human Genetics, Nuffield Department of Medicine, University of Oxford |
| 99 Wellcome Sanger Institute |
| 100 West of Scotland Specialist Virology Centre, NHS Greater Glasgow and Clyde |
